# Current Insights into the Molecular Mechanisms of Intracranial Atherosclerosis and Their Therapeutic Implications

**DOI:** 10.3390/ijms27073266

**Published:** 2026-04-03

**Authors:** Surasak Komonchan, Suchat Hanchaiphiboolkul, Yodkhwan Wattanasen

**Affiliations:** 1 Department of Neuroscience, Bumrungrad International Hospital, Bangkok 10110, Thailand; 2 Department of Neurology, Neurological Institute of Thailand, Bangkok 10400, Thailand; 3 Department of Radiology, Neurological Institute of Thailand, Bangkok 10400, Thailand; slainooo@hotmail.com

**Keywords:** intracranial atherosclerosis, endothelial dysfunction, NLRP3 inflammasome, mechanotransduction, VSMC phenotypic switching, RNF213 variant, neurovascular unit (NVU), vulnerable plaque, precision medicine

## Abstract

Intracranial atherosclerosis (ICAS) is a distinct, inflammation-dominant vasculopathy and a leading cause of global stroke morbidity. Unlike extracranial atherosclerosis (ECAS), which often utilizes compensatory positive remodeling to maintain patency, ICAS is characterized by a unique architecture and a localized antioxidant gap that favor maladaptive negative remodeling. We critically analyze the molecular cascade initiated by the breakdown of the Piezo-type mechanosensitive ion channel component 1 (PIEZO1) and the Krüppel-like factor 2/4 (KLF2/4) mechanotransduction axis, which triggers endothelial nitric oxide synthase (eNOS) uncoupling and establishes a state of chronic inflammation. This environment facilitates the subendothelial lipid retention of oxidized low-density lipoprotein (oxLDL), a process exacerbated by the intracranial deficiency of Apolipoprotein A-I (ApoA-I) and impaired glymphatic clearance. Crucially, we evaluate how these metabolic and mechanical insults drive vascular smooth muscle cell (VSMC) phenotypic switching; the transdifferentiation of contractile VSMCs into macrophage-like foam cells accounts for up to 60% of the plaque’s lipid-laden pool and destabilizes the fibrous cap. This vascular failure directly compromises the neurovascular unit (NVU), leading to pericyte dropout and blood–brain barrier breakdown. Beyond environmental stressors, we highlight the ring finger protein 213 (RNF213) variant as a critical genetic determinant of this susceptibility. Shifting the clinical paradigm from simple luminal narrowing toward the identification of the vulnerable plaque, we discuss how High-Resolution Vessel Wall Imaging (HR-VWI) and microRNA biomarkers can identify unstable lesions. By integrating these molecular and imaging signatures, we propose a precision medicine framework centered on the NLR family pyrin domain containing 3 (NLRP3) inflammasome and the NVU to effectively mitigate the high residual recurrence risk that persists under conventional therapy.

## 1. Introduction

Intracranial atherosclerosis (ICAS), characterized by the progressive accumulation of atherosclerotic plaques within the major arteries at the base of the brain, is a primary driver of global ischemic stroke. Despite its prevalence, ICAS is often considered a mere subset of systemic atherosclerosis, a view that may overlook the unique histological and molecular landscape of the cerebral vasculature. Unlike extracranial arteries, intracranial vessels possess a thin adventitia and a lack of basal vasa vasorum. These structural constraints create a localized antioxidant gap, stemming from a deficiency in protective enzymes like superoxide dismutase (SOD), leaving the neurovascular unit uniquely vulnerable to chronic oxidative stress and impaired metabolic repair.

At the molecular level, this vulnerability is driven by a profound failure of cellular mechanotransduction. Central to ICAS pathogenesis is the disruption of the PIEZO1 KLF2/4 axis which responds to disturbed hemodynamic shear stress and shifts the endothelium into a proinflammatory state. This transition triggers a cascade of subendothelial lipid retention and the activation of the NLRP3 inflammasome, facilitated by impaired glymphatic clearance. Crucially, these molecular insults drive vascular smooth muscle cell (VSMC) phenotypic switching; the transdifferentiation of contractile VSMCs into macrophage-like foam cells destabilizes the fibrous cap and compromises the integrity of the blood–brain barrier.

The unrelenting nature of this process is also evident in the rising prevalence of premature ICAS (ages 18 to 45) [1]. In these cases, the disease is frequently accelerated by the RNF213 variant, a key genetic determinant that exacerbates vascular wall breakdown under mechanical stress. Even with intensive medical therapy consisting of dual antiplatelets and high intensity statins, patients face a significant residual inflammatory risk that maintains high recurrence rates. This review explores the molecular transitions that establish ICAS as a distinct, inflammation dominant vascular disease. By establishing a brain specific molecular framework, we aim to identify recalcitrant therapeutic targets beyond conventional systemic management that are currently on the horizon.

## 2. Materials and Methods

### 2.1. Search Strategy and Information Sources

The methodology for this study was developed using a dual framework approach, combining the PRISMA 2020 structural guidelines with a State of the Art (SotA) interpretive synthesis. To ensure a comprehensive and current evidence base, a systematic search was executed across PubMed, Scopus, and Web of Science, as these databases provide the most robust coverage of high impact biomedical and molecular biology literature. The search period included literature published between January 2010 and February 2026.

The search strategy utilized targeted Boolean strings to identify the intersection of molecular biology and clinical outcomes. Keywords used in the search included: intracranial atherosclerosis, ICAS, intracranial arterial stenosis, molecular mechanisms, pathogenesis, therapeutic resistance, recalcitrant, suboptimal response, and standard of care. This search was designed to specifically capture the wide breadth of evidence regarding the unique molecular landscape of intracranial arteries distinguishing them from systemic and extracranial vessels while identifying studies that discuss the mechanical and biological limitations of current clinical interventions.

### 2.2. Study Selection and SotA Synthesis

Following the PRISMA framework, identified records were manually screened for duplicates and relevance. From an initial pool of literature identified through our search strategy, a total of 92 relevant studies were included in the final synthesis based on their technical quality and clinical relevance. Both authors independently performed a screening of titles and abstracts, followed by a rigorous full text eligibility assessment. Any discrepancies in study selection were resolved through a formal collaborative discussion and consensus between the three authors.

Inclusion criteria were limited to:

- Original research exploring molecular pathways such as inflammation, lipid metabolism, and endothelial dysfunction.
- Clinical trials or meta analyses evaluating outcomes of standard of care treatments, with a specific focus on long term follow up data such as CASSISS 2025 [2] and SAMMPRIS long term outcomes.
- Mechanistic reviews providing high level synthesis of ICAS biology.

Exclusion criteria were applied to case reports without molecular data, non-English publications, and studies with insufficient evidence support.

### 2.3. Quality Assessment and Therapeutic Gaps

To address the SotA component, the final included studies were categorized based on their contribution to identifying emerging therapeutic gaps and recalcitrant disease phenotypes. This categorization allowed for an objective and evidence-based analysis of why certain ICAS cases demonstrate a lack of clinical response to conventional therapy. By prioritizing high impact peer reviewed molecular data and recent comprehensive studies from 2024 to 2026, the review moves from a purely descriptive summary to a mechanistic evaluation of therapeutic implications.

This methodology ensures that the discussion of novel targets such as selective NLRP3 inhibition and RNF213 targeted pathways is grounded in a systematic evaluation of validated molecular signatures. Furthermore, the synthesis specifically highlights studies that demonstrate a residual inflammatory risk, providing a structural framework to explain the 14.3 percent recurrence rate observed in the latest long-term clinical follow up data.

## 3. Epidemiology and Pathophysiological Determinants of the Intracranial Vasculature

Intracranial atherosclerosis (ICAS) represents a significant global healthcare burden characterized by striking geographic and ethnic disparities. It accounts for 30 to 50 percent of ischemic strokes in Asian populations, compared to only 5 to 10 percent in Caucasian cohorts [3]. While the prevalence of ICAS increases steeply with age, affecting approximately 7 percent of asymptomatic middle-aged individuals and rising to 50 percent by the eighth decade [4], this phenotype is also increasingly notable in younger demographics. Premature ICAS in adults aged 18 to 45 is now recognized as a predominant cause of intracranial stenosis in young Chinese populations. Recent multicenter studies and registries from 2024 and 2026 confirm that ICAS remains the most frequent cause of stroke in East Asia despite advancements in primary prevention. These ethnic disparities persist even in younger cohorts; while ICAS accounts for 11.8 percent of strokes in young Western patients, it is diagnosed in 27.4 percent of young Asian patients, often appearing in the absence of traditional risk factors [1]. This disproportionate burden is increasingly linked to specific genetic predispositions, such as the RNF213 p.R4810K variant, which is detected in up to 24 percent of Japanese patients with intracranial stenosis. By regulating vascular endothelial function and angiogenesis, this variant serves as a critical genetic driver of early-onset ICAS [5].

Complementing these genetic factors, the susceptibility to ICAS is further predicated on a unique vascular architecture that distinguishes the intracranial environment from the rest of the systemic circulation. Intracranial arteries are characterized by a denser internal elastic lamina and a thinner tunica media with a relative paucity of vasa vasorum. Contemporary data published after 2021 [6,7,8,9] emphasize that this structural environment creates a localized antioxidant gap, as the lack of a dedicated microvascular supply limits the vessel’s compensatory and reparative capacity when the endothelium is insulted by high pulsatile strain. This gap represents a critical biochemical threshold; during the third and fourth decades of life, intracranial arteries utilize local stores of superoxide dismutase (SOD) and glutathione peroxidase to neutralize hemodynamic-induced oxidative stress. However, while extracranial vessels can replenish these enzymes through vasa vasorum-mediated systemic delivery, the intracranial wall relies on a finite local supply that often becomes depleted by the end of the fourth decade. This enzymatic exhaustion leaves the subendothelial space defenseless against the accumulation of reactive oxygen species (ROS), explaining why ICAS often manifests as a rapidly progressive vasculopathy once this antioxidant capacity is lost [9,10].

These histological vulnerabilities create a perfect storm for high recurrence rates that challenge current clinical standards. While the overall 12-month recurrence rate in general clinical practice is approximately 5.99 percent, this risk remains unacceptably high in symptomatic high-grade subgroups. Historically, the SAMMPRIS trial demonstrated a residual 12-month recurrence risk of 12.2 percent despite intensive medical therapy. In contemporary high-risk cohorts with 70 to 99 percent stenosis, recurrence rates can escalate to between 14.8 percent and 19.0 percent [11]. This persistent failure suggests that standard intensive therapy, while effective at managing systemic risk factors, does not adequately address the unique local environment of the intracranial wall. Conventional statins and antiplatelets primarily target systemic lipid levels and global platelet aggregation, yet they leave behind a significant residual risk within the cerebral vasculature [2,12]. This therapeutic gap is likely driven by localized molecular barriers, including impaired metabolic clearance and chronic mechanical stress, that persist despite optimal medical management. Ultimately, this failure underscores the necessity of moving beyond systemic management toward targeted molecular interventions.

## 4. Topographic Distribution and Stroke Pathophysiology

The clinical manifestation of ICAS is inextricably linked to its anatomical distribution. In Asian cohorts, the disease follows a characteristic pattern where the middle cerebral artery (MCA) is the primary hotspot, accounting for the highest proportion of symptomatic cases [1,4]. This is followed in frequency by the supraclinoid segment of the internal carotid artery (ICA) and the intracranial vertebral artery (VA). Within the posterior circulation, the VA and basilar artery (BA) represent more frequent sites of involvement compared to the posterior cerebral artery (PCA). In contrast, the anterior cerebral artery (ACA) remains the least common site for isolated atherosclerotic disease. These distribution patterns carry profound clinical weight, as the risk of stroke recurrence is significantly higher when severe stenosis or occlusion involves multiple territories [13].

Beyond simple prevalence, specific segments exhibit unique biological vulnerability based on their architecture. The cavernous ICA, for instance, is characterized by a specialized ‘S-shaped’ siphon geometry. The distinct curvature and bending angles of its posterior and anterior bends create a complex hemodynamic environment; this results in low wall shear stress and a high oscillatory shear index, which together facilitate lipid deposition and plaque formation [14]. This geometric configuration forces the blood flow to strike the outer walls of the curves while creating areas of stagnant flow on the inner curves, effectively trapping low-density lipoproteins (LDL) and inflammatory cells against the endothelium. This S-shaped vulnerability underscores why the cavernous segment remains a frequent site for high plaque burden even in patients with well-controlled systemic lipids.

Importantly, the risk of stroke in ICAS often dissociates from the degree of luminal stenosis [10]. Emerging evidence from intracranial vessel wall MR imaging (VWI) suggests that symptomatic plaques are strongly associated with a higher plaque burden, defined as the total cross-sectional area of the vessel wall occupied by the lesion and the presence of intraplaque hemorrhage. While intraplaque hemorrhage is less frequent in the intracranial circulation than in the carotids, it serves as a critical marker of plaque instability and a potent predictor of downstream ischemic events [15], even when the lumen itself appears relatively preserved. Evidence suggests that the hemodynamic state across a lesion, specifically the transition from high velocity pre-stenotic flow to turbulent post-stenotic flow and the resulting pressure gradients, is more predictive of clinical events than the percentage of narrowing alone [6].

These factors converge into four distinct pathophysiological pathways, which frequently overlap, allowing multiple mechanisms to contribute to a single clinical event [16]:

- Artery-to-Artery Embolism: This occurs when a vulnerable atherosclerotic plaque even one causing only mild stenosis, undergoes surface erosion or rupture, releasing thrombi that causes distal embolization.
- Hemodynamic Failure: This mechanism is typically associated with high-grade stenosis or total occlusion, where impaired clearance of emboli (washout) and reduced flow cannot meet metabolic demand, often resulting in “border-zone” or watershed infarcts.
- Branch Atheromatous Disease (BAD): A hallmark of ICAS where the parental artery plaque extends to or occludes the ostia of small perforating arteries (such as the lenticulostriate or paramedian pontine arteries), leading to single subcortical infarctions.
- In situ Thrombotic Occlusion: Acute thrombus formation occurs directly at the site of the atherosclerotic plaque, which can lead to sudden total occlusion of the parent vessel.

## 5. Histopathological and Biological Divergence: ICAS vs. ECAS

The clinicopathological signatures of intracranial (ICAS) and extracranial atherosclerosis (ECAS) diverge significantly, reflecting the unique structural and hemodynamic environments of their respective circulations. While sharing a common systemic origin, the vessel wall architecture dictates the nature of the plaque. ECAS typically follows the Glagovian model of compensatory expansive remodeling, which delays luminal narrowing but is frequently associated with large lipid rich necrotic cores and thin cap fibroatheromas [17]. A defining feature of the extracranial carotid is a robust adventitia containing a network of vasa vasorum present at birth, facilitating extensive neovascularization and a high prevalence of intraplaque hemorrhage.

Intracranial arteries, however, are predicated on a fundamentally different structural paradigm. The most significant divergence is the presence of a denser, reinforced internal elastic lamina (IEL). While this architecture is an evolutionary adaptation to withstand the high pulsatile strain of cerebral blood flow, it serves as a deleterious facilitator in atherogenesis. Because the IEL is significantly thicker and lacks the fenestrations found in systemic vessels, it creates a unidirectional barrier for lipids; once low-density lipoproteins (LDL) penetrate the endothelium, the dense IEL sequestrates them within the subendothelial space, preventing their outward clearance and accelerating the formation of pathological intimal thickening (PIT) [18].

This metabolic entrapment is exacerbated by the relative absence of a tunica adventitia and the lack of basal vasa vasorum. In systemic vessels like the carotid, a thick adventitia provides a structural conduit for metabolic waste clearance and an entry point for oxygenation through the vasa vasorum. The intracranial vessel, by contrast, is a paucity of structural components that lacks this external microvascular network. As the plaque progresses from adaptive intimal thickening to PIT, the diffusion distance from the lumen to the media increases. Without an adventitial vasa vasorum to bridge this gap, the thickened vessel wall enters a state of chronic metabolic compromise.

This structural nutrient deprivation forces the vessel to develop acquired neovascularization, a reactive compensatory response where immature, leaky microvessels sprout to supply the hypoxic tissue [10,19]. On high resolution MRI (VWI), this is visualized as prominent arterial wall enhancement as gadolinium contrast leaks into the plaque matrix. This process explains why ICAS plaques transition from stable, proliferative fibrous lesions into unstable, high-risk phenotypes. Despite these vulnerabilities, the cellular, proteoglycan rich nature of ICAS allows for higher biological plasticity, which explains why ICAS regression rates under aggressive medical therapy (14–28%) are significantly higher than those seen in the fixed, calcified plaques of the extracranial circulation [20].

Consequently, the mechanisms underlying symptomatic events in ICAS diverge fundamentally from those in ECAS (Table 1). While ECAS instability is often driven by neovascular leakage and hemorrhage originating from the adventitial microvasculature, symptomatic ICAS more frequently results from luminal micro fissures. These represent focal disruptions in the fibrous cap that facilitate the centripetal entry of blood from the lumen into the plaque matrix [21]. This luminal driven mechanism of rupture reflects the mechanical vulnerability of the intracranial wall under high pulsatile strain. Despite these risks, ICAS exhibits superior biological plasticity compared to systemic atherosclerosis. The cellular and proteoglycan rich matrix of ICAS plaques remains responsive to metabolic modulation, which explains why regression rates under aggressive medical therapy (14–28%) significantly exceed those of the fixed, calcified plaques found in the extracranial circulation [20].

## 6. Remodeling Dynamics and Morphological Eccentricity

The structural evolution of ICAS is distinguished by remodeling patterns that often deviate from the predictable expansive remodeling observed in the extracranial carotid [21]. These patterns are governed by mechanotransduction, a process where cells convert mechanical flow into biological signals. Specifically, areas of low and oscillatory fluid shear stress at inner curvatures and bifurcations trigger a molecular signature that alters matrix metalloproteinase expression and promotes inward remodeling [22]. In the intracranial circulation, the majority of lesions exhibit an eccentric morphology, with an eccentric distribution appearing in approximately 65% to 70% of cases within the middle cerebral artery (MCA) and basilar artery (BA). The clinical significance of this eccentricity lies in its spatial relationship to perforating branches. For instance, when eccentric lesions in the MCA involve the superior wall where the lenticulostriate arteries originate, they provide the anatomical substrate for Branch Atheromatous Disease (BAD). This positioning allows the parent artery plaque to physically occlude the perforator ostia, leading to small deep infarcts [23,24]. Ultimately, the progression of these lesions is dictated by two divergent remodeling responses: positive remodeling, which involves an initial outward expansion to accommodate the plaque, and negative remodeling, which results in luminal narrowing and increased stroke risk.

The structural evolution of ICAS is distinguished by its remodeling patterns, which often deviate from the predictable expansive remodeling seen in the extracranial carotid [21]. Atherosclerotic plaques frequently develop at the inner curvatures of arteries, branchpoints, and bifurcations. These areas are defined by low and oscillatory fluid shear stress that favor local vascular inflammation and lesion growth [22]. Understanding these patterns is critical, as remodeling is governed by mechanotransduction, the process by which cells convert mechanical flow into biological signals. While high fluid shear stress typically stimulates the endothelium to release nitric oxide and promote outward vessel maintenance, areas of low flow trigger a different molecular signature that alters matrix metalloproteinase expression and promotes inward remodeling.

In the intracranial circulation, the majority of lesions exhibit an eccentric morphology, with an eccentric distribution in approximately 65% to 70% of cases within the middle cerebral artery (MCA) and basilar artery (BA). The clinical significance of this eccentricity lies in its spatial relationship to perforating branches. In the MCA, for instance, plaques predominantly involve superior or ventral walls; when these eccentric lesions encroach upon the superior wall where the lenticulostriate arteries originate, they provide the anatomical substrate for Branch Atheromatous Disease (BAD). This junctional positioning allows the parent artery plaque to physically occlude the perforator ostia, leading to small deep infarcts [23,24].

The progression of these lesions is dictated by two divergent remodeling responses:

- Positive Remodeling: As the initial response to plaque formation, positive remodeling is driven by the localized expression of matrix metalloproteinases, specifically MMP-2 and MMP-9, which enzymatically degrade the extracellular matrix to facilitate outward expansion [7]. However, the unique architecture of intracranial vessels, specifically the dense internal elastic lamina, often resists this expansive force more vigorously than systemic arteries. This mechanical resistance leads to a partially compensated state where, in the early stages of ICAS, the vessel may successfully maintain a normal luminal diameter. This effectively masks the underlying disease on traditional digital subtraction angiography [25,26]. Yet, because expansion is limited by the stiff intracranial wall, the plaque is forced to develop high-risk features, such as larger lipid cores and elevated inflammatory cytokines (e.g., TNF-α and IL-6), within a highly constrained space [7,27]. Consequently, this incomplete expansion creates a deceptive clinical picture where a vessel appears non-stenotic despite harboring a high-volume, unstable plaque. This explains why embolic strokes can occur in patients with less than 50% narrowing; the plaque has grown outward enough to hide the stenosis but not enough to stabilize the inflammatory core.
- Negative Remodeling: More characteristic of the fibrous subtype of ICAS, this process is defined by a shrinkage or constriction of the outer vessel wall [26,27]. This behavior is particularly deleterious as it prevents the outward displacement of the plaque and forces the total plaque burden into the luminal space, leading to rapid symptomatic narrowing. While these plaques are typically more structurally stable and carry a lower risk of rupture, they are the primary drivers of hemodynamic failure and Branch Atheromatous Disease (BAD) due to the uncompensated decrease in vessel diameter [26,27,28].

These divergent remodeling patterns represent the macroscopic conclusion of a process that begins at the cellular level [29]. To understand why the vessel wall initiates these structural shifts, it is necessary to examine endothelial dysfunction, the primary molecular event that disrupts vascular homeostasis and sets the stage for ICAS progression.

## 7. Endothelial Dysfunction: The Initiating Molecular Event

### 7.1. The Anatomical Vulnerability of the Intracranial Endothelium

Endothelial dysfunction represents the earliest and most critical molecular event in the pathogenesis of ICAS. Unlike systemic atherosclerosis, where the vasa vasorum plays a primary role in lipid delivery, ICAS development is uniquely dependent on an intrinsic endothelial molecular shift. The relative absence of vasa vasorum in intracranial vessels places the metabolic burden of homeostasis almost entirely on the endothelial KLF2/4 axis [30]. In its physiological state, the endothelial glycocalyx, a dense, gel-like meshwork of membrane-bound proteoglycans and glycoproteins, serves as the primary homeostatic shield of the intracranial vessel. This layer acts as both a mechanical buffer and a signal transducer that transmits the physical hydrodynamic forces of blood flow to the cytoskeleton to maintain nitric oxide production. Crucially, the enzymatic degradation of this glycocalyx represents a significant early pathological shift in ICAS that precedes visible plaque formation [9]. This erosion is initiated by low or oscillatory wall shear stress, which triggers the upregulation of heparanase and metalloproteinases that cleave the protective glycosaminoglycan chains. This loss of the endothelial surface impairs the vessel’s capacity for mechanosensing, thereby disrupting the mechanotransduction pathways required to maintain vascular tone and directly facilitating the subendothelial retention of low-density lipoproteins [31].

### 7.2. The Integrated Mechanosensory Complex

**Figure 1:**
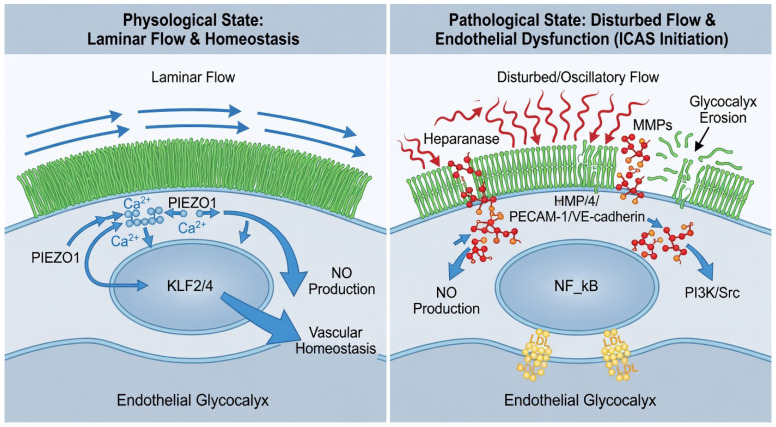
Endothelial Dysfunction and the Initiation of ICAS. Showing the transition from a Physiological State of laminar flow to a Pathological State characterized by disturbed flow. The (**left**) panel illustrates how laminar flow maintains the PIEZO1-KLF2/4 axis and a healthy endothelial glycocalyx. The (**right**) panel depicts the erosion of the glycocalyx by heparanase and MMPs, leading to the nuclear translocation of NF-κB and the creation of a subendothelial sequestration niche for lipoproteins.

Intracranial arteries are also continuously exposed to high pulsatile strain, rendering endothelial cells acutely sensitive to hemodynamic perturbations. Central to this sensing mechanism is the PIEZO1 mechanosensitive ion channel, which acts as the primary transducer of frictional shear stress into intracellular calcium signaling. Beyond PIEZO1, the endothelial junctional complex, comprising PECAM-1, VE-cadherin, and VEGFR2, serves as a vital mechanosensory unit. In the presence of disturbed flow, this complex triggers the activation of the PI3K and Src signaling pathways, which subsequently promote the nuclear translocation of NF-kB and the upregulation of pro-inflammatory adhesion molecules. This junctional sensing works in coordination with integrin-mediated mechanotransduction, where the mechanical tension between the extracellular matrix and the internal cytoskeleton triggers focal adhesion kinase (FAK) activation. These integrated sensors ensure that the endothelial cell can respond to both shear stress and cyclic stretch, creating a coordinated pro-atherogenic response when flow is disturbed. Under physiological laminar flow, PIEZO1-mediated calcium influx triggers the nuclear translocation of flow-responsive transcription factors, KLF2 and KLF4 [30,32], which maintain vascular homeostasis by upregulating nitric oxide production and stabilizing endothelial barrier integrity. However, the early loss of the glycocalyx antenna effectively strips the endothelium of its mechanicalsensor, causing the vessel to lose these protective KLF signals and become porous to systemic lipids (Figure 1).

### 7.3. The Synergistic Multifactorial Insult: Mechanical and Environmental Stressors

This mechanotransduction axis is frequently compromised by a synergistic multifactorial insult of systemic and environmental stressors. While chronic hypertension and pulsatile strain provide the primary mechanical insult, metabolic disturbances and environmental toxins act as potent biochemical catalysts. Notably, fine particulate matter (PM 2.5) air pollution has emerged as a prevalent and decisive risk factor in many Asian regions where ICAS incidence is highest [33]. These pollutants, along with cigarette smoke, enter the systemic circulation and act as potent triggers for vascular inflammation. These diverse insults converge on the activation of NADPH Oxidase (NOX-2 and NOX-4 isoforms), generating a surge of reactive oxygen species that leads to eNOS uncoupling [8]. In this uncoupled state, eNOS shifts from nitric oxide production to superoxide generation [9], a transition exacerbated by the unique antioxidant gap of intracranial vessels. Unlike systemic arteries, intracranial vessels exhibit lower constitutive expression of protective enzymes such as SOD-1, SOD-2, and Glutathione Peroxidase [31]. This localized environment of oxidative stress accelerates lipid oxidation and vascular breakdown.

### 7.4. Transcriptional Shifting and the Creation of a Sequestration Niche

Subsequently, this pro-oxidative environment triggers endothelial activation by disrupting the critical balance between the KLF factors and NF-kB [34]. In the disturbed flow patterns typical of ICAS-prone sites, where flow becomes oscillatory and turbulent, the loss of KLF-mediated inhibition allows for the rapid nuclear translocation of NF-kB. This transcriptional hub translates mechanical and oxidative stress into a coordinated pro-inflammatory program, characterized by the upregulation of adhesion molecules (VCAM-1, ICAM-1, and E-selectin) and the secretion of chemokines like MCP-1 [34,35]. Concurrently, this signaling disrupts tight junction proteins and promotes cytoskeletal reorganization, increasing endothelial permeability. This inflammation-dominant phenotype does not merely alter the endothelial surface but serves as the gateway for the sequestration of lipids. Once the barrier is compromised and the glycocalyx is stripped, the intracranial wall transitions from a homeostatic barrier into a sequestration niche for lipoproteins, establishing the chronic inflammatory cascade that fundamentally distinguishes ICAS from its extracranial counterparts.

## 8. Lipid Retention and Impaired Reverse Cholesterol Transport

The compromise in endothelial barrier integrity facilitates the influx of low-density lipoproteins into the subendothelial space, where they are sequestered via binding to extracellular matrix proteoglycans [36]. Within this pro-oxidative environment, these lipoproteins undergo oxidative modification into oxidized LDL, a high-affinity ligand for macrophage scavenger receptors CD36 and SR A [37].

Crucially, this lipid accumulation triggers a failure in Reverse Cholesterol Transport, the primary mechanism for plaque regression. While the ATP binding cassette transporters ABCA1 and ABCG1 physiologically export excess cholesterol to HDL acceptors, the intracranial environment dominated by upregulated NF kB and pro inflammatory cytokines such as TNF α and IL 1β suppresses the transcription of these efflux transporters [38,39]. This failure is further exacerbated by the unique lipid biology of the cerebral microenvironment, specifically the predominance of small and dense HDL 3 particles. Unlike the larger HDL 2 isoforms found more abundantly in systemic circulation, HDL 3 exhibits a reduced capacity for cholesterol efflux. This limitation is compounded by the significantly lower constitutive concentrations of Apolipoprotein A-I in intracranial arteries, which fundamentally restricts the availability of primary acceptors for ABCA1 mediated transport.

**Figure 2:**
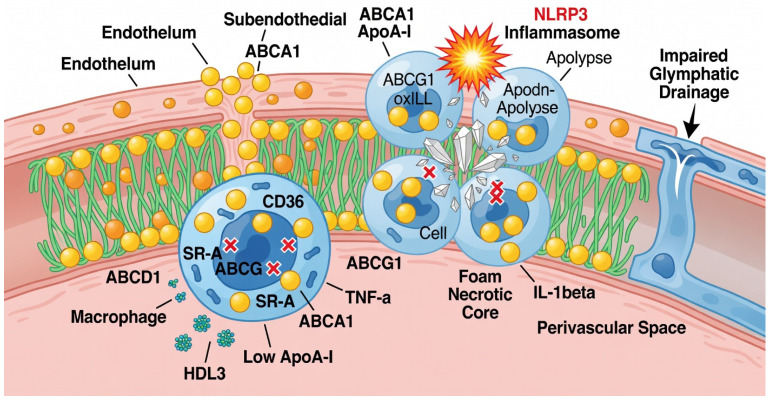
Lipid Retention and Impaired Glymphatic Clearance. Showing the failure of Reverse Cholesterol Transport (RCT) within the intracranial environment. The diagram illustrates how a deficiency in ApoA-I and the predominance of HDL3 impair cholesterol efflux via ABCA1/ABCG1 transporters. On the right, the figure highlights how impaired perivascular glymphatic drainage leads to the localized accumulation of foam cells and the formation of a necrotic core.

Consequently, the inability to export lipids leads to intracellular congestion and the differentiation of macrophages into foam cells. Unlike systemic arteries that utilize a robust adventitial lymphatic network for waste removal, intracranial vessels rely on perivascular glymphatic clearance pathways which are frequently impaired by chronic hypertension and arterial stiffness [31,40]. In the setting of ICAS, this impaired glymphatic drainage leads to the localized entrapment of lipid laden cells. As these cells become overwhelmed, they undergo programmed cell death through apoptosis and necroptosis. Without a lymphatic exit to clear this debris, the resulting cellular remnants aggregate to form a necrotic core [41]. This accumulation of metabolic waste and cholesterol crystals does not remain inert; instead, these endogenous danger signals serve as the primary trigger for the next phase of the inflammatory cascade: the activation of the NLRP3 inflammasome (Figure 2).

## 9. The NLRP3 Inflammasome as a Pathogenic Multiplier

A pivotal molecular driver within this inflammatory milieu is the NLRP3 (nucleotide binding oligomerization domain like receptor family pyrin domain containing 3) inflammasome. In ICAS, the NLRP3 complex acts as a specialized intracellular sensor that responds to Damage Associated Molecular Patterns (DAMPs) ubiquitous in the intracranial plaque, including cholesterol crystals and extracellular ATP released from necroptotic cells [35,42].

Upon activation, the inflammasome serves as a platform for the proteolytic cleavage of pro-caspase 1 [43]. This activation process is heavily dependent on a two-step signaling mechanism where the initial priming is driven by a critical crosstalk between mitochondrial reactive oxygen species ROS and the NLRP3 complex. Specifically, dysfunctional mitochondria in the intracranial vessel wall release oxidized mitochondrial DNA into the cytosol, which acts as a potent secondary signal that stabilizes the NLRP3 assembly and ensures a sustained inflammatory response. This leads to the maturation and secretion of highly potent pro inflammatory cytokines, primarily interleukin 1β (IL 1β) and IL 18, while simultaneously triggering pyroptosis [44], an inflammatory form of programmed cell death.

**Figure 3:**
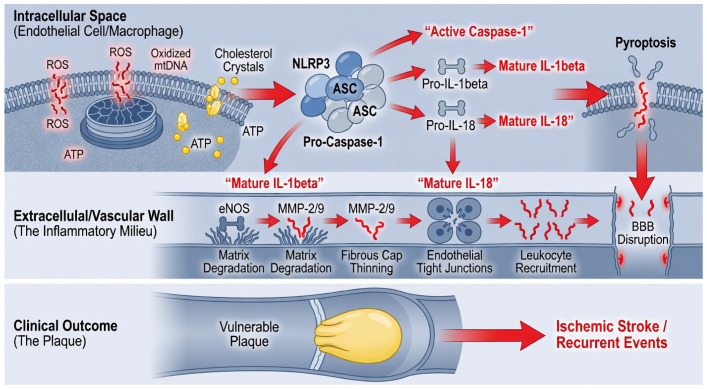
The NLRP3 Inflammasome Pathway and Clinical Outcomes. Showing the signaling cascade from intracellular stress to plaque rupture. The top panel details the activation of the NLRP3 complex, leading to the maturation of IL-1β and IL-18 and the initiation of pyroptosis. The bottom panel correlates this inflammatory milieu with matrix degradation, blood–brain barrier (BBB) disruption, and the formation of a vulnerable plaque that results in ischemic stroke.

While IL 1β is a primary driver of matrix degradation, the concurrent release of IL 18 exerts a profound effect on the neurovascular unit by directly facilitating blood–brain barrier (BBB) disruption. IL 18 signaling increases the permeability of endothelial tight junctions and stimulates the production of chemokines that recruit circulating leukocytes into the perivascular space, thereby bridging vascular inflammation with neural tissue damage. Unlike other cytokines, IL 1β exerts a profound bystander effect, further downregulating eNOS activity and stimulating the production of Matrix Metalloproteinases (MMPs), particularly MMP 2 and MMP 9 [45]. This localized cytokine surge degrades the extracellular matrix and destabilizes the fibrous cap [46], transforming a stable lesion into a high-risk plaque prone to rupture (Figure 3). The clinical relevance of this pathway is supported by evidence from human ICAS plaque samples, which demonstrate a significantly higher concentration of NLRP3 components and mature IL 1β within symptomatic lesions compared to stable plaques. These human data confirm that the intensity of inflammasome activation is a primary determinant of plaque vulnerability and is directly correlated with the risk of recurrent ischemic events. Given the unique anatomical constraints and limited remodeling capacity of intracranial arteries, the NLRP3 IL 1β axis represents a critical therapeutic target [47,48]; its inhibition offers a potential strategy to arrest the inflammatory cascade that leads to symptomatic ICAS.

## 10. Chronic Inflammation and Immune Dysregulation in ICAS

Beyond the initial stages of lipid retention, ICAS is driven by a state of chronic immune dysregulation where innate and adaptive signals converge to dictate plaque instability. Unlike the lipid-heavy paradigms of extracranial disease, the intracranial environment fosters a disproportionately aggressive inflammatory milieu.

### 10.1. The Innate-Adaptive Axis and Polarization Failure

Following transmigration, monocyte-derived macrophages predominantly undergo M1 (pro-inflammatory) polarization, a state reinforced by a Th1-M1 feed-forward loop [49,50]. Infiltrating T-helper 1 (Th1) and Th17 cells secrete Interferon-ɣ (IFN- ɣ) and IL-17, which amplify macrophage production of TNF-α, IL-6 [51], and reactive oxygen species (ROS). This pro-inflammatory axis actively suppresses the alternative M2 (pro-resolving) and regulatory T cell (Treg) phenotypes, which are essential for tissue repair and immune tolerance.

A critical consequence of this M1-biased environment is the profound impairment of efferocytosis, which prevents the timely clearance of apoptotic and necroptotic foam cells, thereby driving secondary necrosis and plaque destabilization [52]. In the intracranial plaque, this defective clearance creates a self-amplifying cycle: as foam cells undergo secondary necrosis, they release Damage-Associated Molecular Patterns (DAMPs) that further aggravate the local environment [53].

### 10.2. NLRP3 Signaling: From Pathogenic Platform to Therapeutic Target

The NLRP3 inflammasome functions as the pivotal molecular sensor linking this cellular debris and lipid accumulation to active vascular destruction. Within the intracranial macrophage population, cholesterol crystals and mitochondrial ROS serve as second signals that trigger caspase-1–mediated cleavage [54] and the release of mature IL-1β and IL-18.

In the context of ICAS, this signaling is particularly detrimental; IL-1β not only amplifies endothelial activation but also stimulates the production of Matrix Metalloproteinases (MMPs) that degrade the internal elastic lamina [25]. This sustained immune imbalance ensures the intracranial plaque remains an active site of biological stress, shifting its trajectory toward high-risk vulnerability.

Notably, the NLRP3-IL-1β axis has emerged as a primary therapeutic target to address the residual inflammatory risk that persists despite intensive statin therapy. Clinical evidence from the CANTOS and COLCOT trials has validated that targeting this pathway can reduce vascular events independently of lipid lowering [47,49]. In ICAS, specific NLRP3 inhibitors (e.g., small molecule diarylsulfonylurea derivatives) represent a promising frontier [55] for stabilizing the active molecular phenotype of the vessel wall and preventing the enzymatic degradation that leads to embolic recurrence.

## 11. The Neurovascular Unit and Immune Crosstalk

Inflammation in ICAS extends beyond the focal arterial stenosis, involving dynamic and bidirectional interactions with the components of the Neurovascular Unit (NVU). This structural and functional syncytium comprising endothelial cells, pericytes, astrocytes, and microglia is essential for maintaining the blood–brain barrier (BBB) [56,57] and regulating cerebral blood flow through neurovascular coupling, the functional link between local neuronal activity and corresponding hemodynamic changes.

Endothelial inflammation and the subsequent downregulation of tight junction proteins (e.g., claudin 5 and occludin) disrupt BBB integrity [56]. This failure facilitates a critical crosstalk between infiltrating circulating immune cells and perivascular neural elements. In the setting of chronic hypoperfusion and inflammatory signaling, glial cells undergo profound phenotypic shifts. Specifically, astrocytes transition from a supportive state into an A1 reactive phenotype, which is characterized by the loss of their ability to promote neuronal survival and the acquisition of neurotoxic functions [58]. This is complemented by a shift in microglia from a homeostatic or M2 reparative state toward an M1 pro inflammatory profile.

Cytokines and chemokines released from activated M1 macrophages and Th1/Th17 cells notably TNF α and IL 1β influence perivascular astrocytes and pericytes, triggering a state of reactive astrogliosis and pericyte detachment [58,59]. Quantitative evidence suggests that ICAS associated chronic inflammation leads to significant pericyte dropout, with some models showing up to a 30% to 50% reduction in pericyte coverage in the perivascular spaces adjacent to symptomatic plaques. This loss of cellular support triggers a cascade of vascular leakage, destabilizes vascular homeostasis, and impairs cerebral blood flow autoregulation [56,60].

The clinical correlate of this breakdown is increasingly captured through Dynamic Contrast Enhanced MRI (DCE MRI), which provides a quantitative measure of the vascular permeability index. This metric calculates the rate at which blood borne substances transit across a compromised blood–brain barrier into the surrounding tissue. High values in the territories distal to an intracranial stenosis serve as an imaging marker for severe NVU dysfunction and are predictive of a higher risk for cognitive decline and recurrent stroke.

Collectively, these inflammatory and immune mechanisms establish ICAS as a chronic, immune mediated vascular disease. Persistent immune activation not only drives progressive luminal narrowing but also significantly increases susceptibility to plaque instability and artery to artery embolic events [61]. By engaging the entire NVU, these pathways provide a direct molecular link between chronic vascular inflammation and the catastrophic clinical endpoints of ischemic stroke.

## 12. Vascular Smooth Muscle Cell (VSMC) Phenotypic Switching and Maladaptive Remodeling

Vascular smooth muscle cells (VSMCs) are no longer viewed as terminally differentiated contractile units, but as highly plastic cells that dictate the structural evolution of the intracranial plaque. In ICAS, the transition of VSMCs from a quiescent, contractile phenotype to a synthetic and pro inflammatory state is a central driver of progressive luminal narrowing [62].

### 12.1. The Contractile to Synthetic Transition

Under physiological conditions, VSMCs maintain vascular tone through the expression of contractile proteins, including smooth muscle alpha actin SMA and myosin heavy chain (SM-MHC/MYH11). However, in the setting of chronic endothelial dysfunction and exposure to platelet derived growth factor-BB (PDGF-BB) and oxLDL, VSMCs undergo a profound phenotypic shift [63]. This transition is characterized by the downregulation of contractile markers and the upregulation of genes associated with migration and extracellular matrix ECM production. In the intracranial vasculature, where arteries possess a thinner medial layer and limited outward remodeling capacity, this migration into the intima results in accelerated neointimal hyperplasia and early-stage luminal compromise.

### 12.2. Key Molecular Pathways: PDGF, Notch, and KLF4

**Figure 4:**
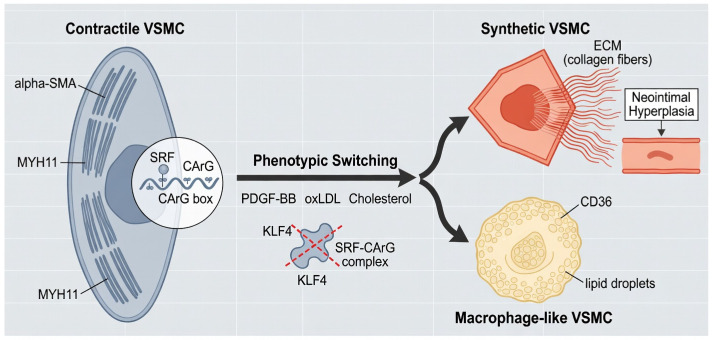
Molecular Mechanisms of VSMC Phenotypic Switching in ICAS. Showing the transdifferentiation of vascular smooth muscle cells (VSMCs) from a contractile stability to an inflammatory synthetic state. The figure illustrates how PDGF-BB and oxLDL trigger the downregulation of contractile markers like alpha-SMA and MYH11. It further shows the transition of VSMCs into macrophage-like cells that express the scavenger receptor CD36, contributing significantly to the plaque’s foam cell population.

The molecular control of VSMC plasticity is governed by several converging axes. Platelet derived growth factor (PDGF) signaling through PDGFR-β activates the MAPK/ERK and PI3K/Akt cascades, driving VSMC proliferation. Simultaneously, the downregulation of contractile genes is mediated by the transcription factor KLF4, which disrupts the binding of Serum Response Factor (SRF) to the CArG box in smooth muscle gene promoters [64]. Developmental pathways, including Notch and Wnt/β-catenin, further modulate VSMC fate; Notch signaling is essential for phenotypic stability [65], while its dysregulation facilitates the transition toward a synthetic state (Figure 4).

### 12.3. Extracellular Matrix (ECM) Remodeling and Macrophage Like Transdifferentiation

Synthetic VSMCs actively remodel the vessel wall by producing collagen and proteoglycans. While initially intended to stabilize the plaque via fibrous cap formation, excessive and disorganized ECM deposition in the intracranial wall reduces arterial compliance and alters local biomechanics. Crucially, emerging quantitative evidence from landmark studies suggests that a significant proportion of foam cells in atherosclerotic lesions are not monocyte derived but are actually SMC derived macrophage like cells [66]. Research has demonstrated that approximately 40% to 60% of foam cells within the plaque are of VSMC origin, appearing after the loss of their traditional myogenic markers and the acquisition of scavenger receptors such as CD36 [67]. Triggered by cholesterol loading, these VSMCs lose their myogenic markers and acquire scavenger receptors, amplifying inflammatory signaling and lipid accumulation. This phenotypic convergence blurs traditional lineage boundaries and identifies the VSMC as a primary contributor to the inflammation dominant nature of intracranial atherosclerosis.

## 13. Plaque Progression, Remodeling, and Thrombo-Inflammatory Complications

Vascular remodeling in ICAS is a dynamic structural process that determines whether an atherosclerotic plaque remains clinically silent or becomes symptomatic. Unlike systemic arteries, where outward expansion can compensate for plaque growth for extended periods, the intracranial vessel wall is constrained by the subarachnoid space and its unique muscular architecture.

### 13.1. Positive Versus Negative Remodeling

Positive remodeling (PR) involves the outward expansion of the vessel wall to maintain luminal patency despite increasing plaque volume. In contrast, negative remodeling (NR) occurs when the vessel wall constricts or fails to expand, leading to accelerated luminal narrowing even with a relatively small plaque burden. In the intracranial vasculature, HR VWI studies have shown that the prevalence of remodeling patterns varies significantly; positive remodeling is identified in approximately 25% to 35% of symptomatic cases, while negative remodeling is more common in stable, chronic lesions. Notably, positive remodeling is strongly correlated with markers of plaque vulnerability, such as high-grade wall enhancement and the presence of intraplaque hemorrhage. These imaging phenotypes help clinicians distinguish between an expansive vulnerable plaque and a constrictive stable lesion, which has direct implications for the risk of acute ischemic events.

### 13.2. The Paradox of Remodeling and Stroke Risk

While positive remodeling may initially preserve blood flow, it often masks the true extent of the atherosclerotic burden and is associated with larger, lipid-rich necrotic cores. Clinical data suggest that lesions demonstrating positive remodeling on HR VWI have a significantly higher association with acute symptomatic stroke compared to those with negative or minimal remodeling. This imaging correlate identifies a high-risk phenotype where the compensatory expansion of the vessel wall fails to stabilize the plaque, leading to rupture or distal embolization. Conversely, negative remodeling in intracranial arteries facilitates rapid luminal compromise but is often associated with more fibrotic, stable plaques. By utilizing these imaging markers to identify specific remodeling phenotypes, we can better predict which patients are at a higher risk of recurrence despite having similar degrees of stenosis on conventional angiography.

Plaque progression in ICAS reflects a dynamic and deleterious interplay between chronic inflammation, cellular apoptosis, extracellular matrix (ECM) remodeling, and thrombogenic signaling. Unlike extracranial atherosclerosis, intracranial plaques are characterized by distinct compositional features, specifically relatively lower calcification rates but significantly heightened inflammatory activity [68,69] which fundamentally drive plaque instability and ischemic risk.

### 13.3. Matrix Degradation and Fibrous Cap Weakening

The structural integrity of the fibrous cap is governed by the balance between ECM synthesis by synthetic VSMCs and proteolytic degradation. Pro-inflammatory cytokines, primarily IL-1β and TNF-α, stimulate macrophages and synthetic VSMCs to overexpress Matrix Metalloproteinases (MMPs), particularly MMP-2 and MMP-9 [46]. These endopeptidases degrade the collagen and elastin fibers that provide the cap’s tensile strength.

In the intracranial environment, the confined anatomy and limited outward (compensatory) remodeling capacity amplify the hemodynamic impact of even minor matrix disruption. This structural weakening predisposes the plaque to surface erosion or rupture, facilitating luminal compromise and downstream embolization.

### 13.4. Necrotic Core Expansion and Defective Efferocytosis

As the plaque matures, the progressive accumulation of apoptotic macrophages and VSMCs leads to the formation of a necrotic core. Under physiological conditions, efferocytosis, the rapid clearance of apoptotic bodies, prevents the transition to secondary necrosis, a state where uncleared apoptotic cells lose membrane integrity and release toxic intracellular contents. However, in ICAS, the localized cytokine surge and oxidative stress impair critical “eat-me” or pro-phagocytic signaling (e.g., the Gas6/MerTK pathway), leading to defective efferocytosis [70].

The resulting secondary necrosis causes the release of intracellular Damage-Associated Molecular Patterns (DAMPs), which further stimulate NLRP3 inflammasome activity and proteolytic expression [53]. This feed-forward inflammatory loop accelerates plaque destabilization and creates a highly pro-thrombotic environment.

### 13.5. The Thrombo-Inflammatory Interface and Stroke Mechanisms

Molecular inflammation in ICAS is inextricably linked to thrombogenic signaling. Endothelial disruption and macrophage activation trigger the expression of Tissue Factor (TF), the primary initiator of the extrinsic coagulation cascade [71]. Concurrently, inflammatory mediators and the loss of endothelial-derived anti-thrombotic factors (like prostacyclin and NO) enhance platelet adhesion and aggregation [72].

These thrombo-inflammatory processes underpin the primary stroke mechanisms in ICAS: artery-to-artery embolism and in situ thrombosis. Crucially, the molecular instability of the plaque, driven by inflammation and matrix degradation, means that symptomatic events can occur even in the absence of high-grade luminal stenosis [72], underscoring the clinical necessity of assessing molecular plaque vulnerability alongside anatomical narrowing.

## 14. Genetic and Epigenetic Determinants of Intracranial Atherosclerosis

Accumulating evidence indicates that intracranial atherosclerosis (ICAS) is not solely a consequence of traditional vascular risk factors but is also strongly influenced by genetic susceptibility and epigenetic regulation. These factors modulate endothelial function, inflammatory responses, and vascular remodeling, thereby shaping individual vulnerability to disease initiation and progression. This genetic contribution is particularly relevant in ICAS, given its marked ethnic and geographic predilection toward East Asian populations [73]. Genome-wide association studies (GWAS) have identified several robust genetic variants, most notably in the *RNF213* gene, which encodes a large E3 ubiquitin ligase. While the p.R4810K variant of *RNF213* is the hallmark of Moyamoya disease, specific polymorphisms in this gene are also linked to non-moyamoya intracranial arterial stenosis [74], suggesting that *RNF213* acts as a vascular frailty gene that sensitizes the intracranial vasculature to hemodynamic and inflammatory insults [75]. Beyond sequence variants, the ICAS phenotype is further sculpted by epigenetic determinants, including DNA methylation and non-coding RNAs, which translate environmental stressors into stable changes in gene expression. Differential methylation of the eNOS and MMP-9 promoters in response to chronic shear stress [76] provides a molecular basis for the localized antioxidant gap and accelerated matrix degradation observed in cerebral vessels. These findings support the concept that ICAS represents a genetically and epigenetically conditioned, inflammation-dominant vascular phenotype. This fundamentally distinguishes it from extracranial atherosclerosis (ECAS), where the genetic architecture and disease trajectory are more heavily weighted toward systemic lipid metabolism [27,77] and traditional large-vessel cholesterol transport mechanisms.

### 14.1. Polygenic Pathways and the Concept of Vascular Memory

Beyond single-gene associations, ICAS is increasingly recognized as a polygenic disorder involving the coordinated dysregulation of vascular and immune networks. Genetic variants influencing endothelial nitric oxide (NO) signaling and oxidative stress responses collectively lower the threshold for endothelial failure. Crucially, these polygenic effects often modulate local plaque biology, such as the intensity of the Th1-M1 inflammatory response, without substantially altering systemic lipid levels [78]. This offers a molecular explanation for the limited efficacy of traditional lipid-focused therapies in certain ICAS populations and underscores why patients with normal LDL levels can still harbor high-volume, unstable intracranial plaques.

### 14.2. Epigenetic Regulation and MicroRNA Signaling

Epigenetic mechanisms provide a dynamic interface between genetic predisposition and environmental exposure. In ICAS, MicroRNAs (miRNAs) have emerged as key post-transcriptional regulators; for example, the depletion of endothelial-enriched miR-126 impairs vascular repair [79], while the upregulation of miR-21 and miR-155 promotes M1-macrophage polarization and cytokine surges [80].

Furthermore, DNA methylation and histone modifications influence transcriptional programs governing chronic inflammatory pathways. These pro-inflammatory epigenetic signatures may persist even after clinical risk factors are controlled, contributing to a state of vascular memory [81,82]. This phenomenon explains why disease activity often persists despite optimal medical therapy, as the vessel wall remains committed to a pro-atherogenic state. Consequently, targeting these epigenetic determinants represents a vital therapeutic frontier for future ICAS interventions, moving beyond simple risk factor management toward the restoration of the vascular epigenome.

## 15. Clinical Translation: High-Resolution Vessel Wall Imaging and Molecular Biomarkers

The clinical management of ICAS is undergoing a paradigm shift from the simple assessment of luminal stenosis toward the precise identification of the vulnerable plaque. High-resolution vessel wall MRI (HR-VWI) has emerged as the gold standard for non-invasively visualizing the molecular processes occurring within the intracranial intima [83]. Specific MRI signatures provide a window into the plaque’s biological state; for instance, gadolinium enhancement on T1-weighted sequences serves as a surrogate marker for active endothelial permeability and M1-macrophage infiltration [83], while intraplaque hemorrhage (IPH), visualized as high-signal intensity, indicates advanced matrix degradation and neovascular failure [84,85]. Unlike extracranial atherosclerosis, ICAS often displays a constrictive or negative remodeling pattern on MRI, reflecting the inflammation-dominant nature and limited outward expansion capacity of intracranial vessels.

To complement neuroimaging, circulating molecular biomarkers provides a systemic liquid biopsy of the intracranial inflammatory burden. Given the antioxidant gap and the Th1-M1 axis previously discussed, plasma levels of hs-CRP, IL-1β, and IL-6 have been strongly correlated with plaque enhancement [86] and a heightened risk of recurrent ischemic events. Furthermore, systemic indicators of matrix turnover, such as the MMP-9/TIMP-1 ratio, reflect the proteolytic activity destabilizing the fibrous cap. Emerging epigenetic markers, specifically circulating miR-155 and miR-21, offer high specificity in reflecting the macrophage polarization state of the plaque [87]. Collectively, the integration of HR-VWI with these molecular signatures enables a bench-to-bedside approach, allowing clinicians to move beyond purely anatomical measurements toward a personalized, molecularly driven risk stratification for ICAS patients.

## 16. Therapeutic Implications and Emerging Molecular Targets

The limited success of conventional strategies focused solely on systemic lipid lowering and antithrombotic therapy [2,12] underscores the need for a shift toward molecularly targeted interventions. The unique, inflammation dominant pathophysiology of ICAS reveals multiple potential intervention points across the disease cascade. The persistent recurrence of the disease despite intensive medical therapy suggests that conventional systemic management fails to address the localized microenvironmental crisis within the intracranial vessel wall. This resistance can be unified under a model of localized homeostatic failure, where the disease is driven by a pathogenic triad of molecular determinants: the intracranial deficit of ApoA-I, mechanosensory switching, and glymphatic stagnation. These three convergent axes represent the current focus for overcoming the limitations of conventional systemic management.

### 16.1. Addressing Localized Homeostatic Failure

While systemic statins and emerging PCSK9 inhibitors effectively lower LDL, they do not correct the localized lack of ApoA I or the predominance of HDL3, which fundamentally restricts the machinery for reverse cholesterol transport. Causal evidence from selective deficiency models confirms that lipid entrapment continues unabated when these localized export pathways are suppressed, regardless of circulating lipid levels. Furthermore, the mechanosensory lock on the NF kB pathway, driven by chronic PIEZO1 signaling, maintains a pro thrombotic endothelial surface that remains active despite systemic antiplatelet therapy. Finally, the impairment of perivascular glymphatic clearance leads to a closed loop of NLRP3 activation, where trapped cholesterol crystals provide a constant inflammatory primer that systemic drugs cannot reach.

### 16.2. Targeting Endothelial Homeostasis and Redox Balance

Restoration of endothelial homeostasis remains a foundational goal. Novel strategies focus on eNOS recoupling agents and selective NOX4 inhibitors to bridge the antioxidant gap [88]. Agents that stabilize the PIEZO1 KLF2 4 axis or mitigate mitochondrial oxidative stress represent a proactive approach to attenuating early disease initiation before the onset of irreversible structural remodeling [89]. However, the therapeutic modulation of PIEZO1 carries significant risks, as this mechanosensor is ubiquitously expressed and essential for maintaining systemic vascular tone and lymphatic function. Nonselective activation or inhibition could lead to unintended consequences such as impaired pressure sensing or disrupted fluid balance, necessitating the development of highly localized delivery systems such as nanoparticle mediated targeting.

### 16.3. Immunomodulatory and Anti-Inflammatory Strategies

As a chronic immune-mediated inflammatory state, ICAS is uniquely suited for immunomodulation. The NLRP3 IL 1β axis is a primary target; clinical data from trials such as CANTOS have validated that inhibiting IL 1β (e.g., via Canakinumab) or the NLRP3 inflammasome (e.g., via MCC950) reduces vascular events independent of LDL levels. Furthermore, shifting the M1 M2 macrophage balance through PPAR ɣ agonists or enhancing regulatory T cell (Treg) function offers a path toward resolving chronic inflammation and promoting active plaque stabilization [90]. Despite this promise, a major limitation for ICAS therapy is the challenge of blood–brain barrier penetration. Many high molecular weight monoclonal antibodies and polar small molecules demonstrate poor central nervous system bioavailability, which may limit their efficacy in stabilizing the perivascular neurovascular unit.

### 16.4. Augmenting Glymphatic Flow and NLRP3 Resolution

To address glymphatic stagnation, emerging therapies focus on restoring perivascular fluid dynamics to flush inflammatory primers like cholesterol crystals. Putative AQP4 facilitators (e.g., TGN-073) or strategies to enhance perivascular AQP4 polarization represent the cornerstone of this strategy, as they restore the convective exchange between CSF and interstitial fluid. This clearance is critical to breaking the closed loop of NLRP3-IL-1β activation. Shifting the M1/M2 macrophage balance through PPAR ɣ agonists further aids in resolving mural inflammation [90], though BBB penetrance of polar small molecules remains a significant hurdle.

### 16.5. RNA Based Therapies and Precision Medicine

The emergence of RNA based therapeutics, including antisense oligonucleotides (ASOs) and small interfering RNAs (siRNAs), allows for the precise modulation of disease driving pathways [91]. Targeting pathogenic miR 155 or enhancing miR 126 expression could theoretically reset the vascular memory of the intracranial endothelium [92]. However, the unresolved biology of the *RNF213* gene presents a significant hurdle. Since the exact enzymatic functions and substrates of the RNF213 E3 ubiquitin ligase are not yet fully understood, targeting this pathway carries the risk of disrupting essential vascular repair mechanisms or angiogenesis.

To effectively mitigate the high residual recurrence risk in ICAS, molecularly targeted therapies must be supported by a comprehensive strategy of synergistic risk modification that restores the localized homeostatic environment of the intracranial wall. Tight blood pressure management and regular aerobic exercise are critical to restoring physiological laminar flow, which promotes the activation of the PIEZO1-KLF2/4 axis, the primary homeostatic shield required to maintain nitric oxide production and endothelial barrier integrity. Furthermore, eliminating cigarette smoke and reducing exposure to environmental toxins like PM 2.5 is a prerequisite for closing the localized antioxidant gap, as these stressors drive eNOS uncoupling and accelerate the enzymatic exhaustion of local SOD stores. Complementing these efforts, optimizing glycemic levels prevents the accelerated modification of trapped lipoproteins into oxLDL, the primary ligand for NLRP3 inflammasome activation. Finally, managing arterial stiffness and chronic hypertension is essential to support perivascular glymphatic clearance, flushing the sequestration niche of trapped cholesterol crystals and breaking the closed-loop activation of the NLRP3-IL-1β axis (Table 2).

Ultimately, the future of ICAS management lies in precision medicine. By integrating *RNF213* genetic profiling, epigenetic biomarkers, and high-resolution vessel wall MRI, clinicians can move beyond the measurement of luminal stenosis toward a personalized strategy that stratifies patients based on their specific molecular and inflammatory fingerprint. This shift prioritizes localized molecular stabilization over nonspecific systemic management to overcome the inherent causes of therapeutic resistance.

## 17. Conclusions and Future Perspectives

Intracranial atherosclerosis (ICAS) is increasingly recognized not as a mere extension of systemic vascular disease, but as a distinct, inflammation-dominant clinical entity shaped by unique anatomical and molecular constraints. The absence of a robust vasa vasorum and a limited antioxidant buffering capacity renders the intracranial vasculature exceptionally dependent on the PIEZO1-KLF2/4 mechanotransduction axis. As this paper has explored, the breakdown of this homeostatic barrier, driven by the synergistic multi-factorial insult of systemic stressors and genetic predispositions like RNF213 variants, triggers a self-amplifying cascade of eNOS uncoupling, M1 macrophage polarization, and NLRP3 inflammasome activation.

The transition of ICAS management into the era of precision medicine depends on our ability to bridge the gap between these microscopic molecular shifts and macroscopic clinical observations. The integration of High-Resolution Vessel Wall Imaging (HR-VWI) with specific circulating biomarkers, such as microRNA signatures and matrix turnover indicators, offers a promising framework for identifying the vulnerable plaque before the onset of catastrophic ischemic events.

Future therapeutic efforts must move beyond systemic lipid-lowering to address the vascular memory imprinted through epigenetic modifications. Emerging strategies, including selective NOX inhibitors, IL-1β antagonists, and RNA-based therapies, provide a roadmap for stabilizing the intracranial vessel wall with unprecedented specificity. Ultimately, shifting the paradigm from reactive stroke prevention to proactive, molecularly driven vascular stabilization holds the potential to significantly reduce the global burden of this high-risk vasculopathy.

## Figures and Tables

**Table 1:** Molecular and Structural Comparison of ICAS and ECAS.

Feature	Intracranial Atherosclerosis (ICAS)	Extracranial Atherosclerosis (ECAS)
Histology	Dense internal elastic lamina; thin media; lack of basal vasa vasorum.	Prominent elastic fibers; robust adventitia; early vasa vasorum presence.
Remodeling	Predominantly negative (constrictive) remodeling.	Glagovian compensatory expansive remodeling.
Plaque Type	Proliferative and fibrous; low calcification.	Lipid-rich necrotic core (LRNC); high calcification.
Pathogenesis	Driven by low wall shear stress and local inflammation.	Driven by systemic lipid metabolism and systemic inflammation.

**Table 2:** Emerging Molecular Targets and Therapeutic Frontiers in ICAS.

Target Axis/Molecule	Pathogenic Role and Molecular Mechanism	Therapeutic Goal and Current Horizon
NLRP3–IL-1β	Acts as an intracellular sensor for cholesterol crystals and DAMPs; drives the maturation of pro-inflammatory cytokines and triggers pyroptosis.	Targeted inhibition of residual inflammatory risk using small molecules (e.g., MCC950) or monoclonal antibodies (e.g., Canakinumab) [47,49,55].
PIEZO1–KLF2/4	Transduces mechanical shear stress into (*Ca*^2+^) signaling; loss of this axis leads to eNOS uncoupling and endothelial barrier breakdown.	Pharmacological stabilization of mechanosensitive signaling to restore KLF2/4 expression and prevent pro-oxidative vascular remodeling [89].
RNF213 Variant	Encodes an E3 ubiquitin ligase acting as a vascular frailty gene; primary susceptibility factor for early-onset ICAS in Asian populations.	Personalized risk stratification via genetic profiling and investigation of targeted angiogenesis modulation strategies.
NOX4 (NADPH Oxidase)	Primary driver of ROS production in the intracranial antioxidant gap, leading to localized oxidative damage and lipid oxidation.	Development of selective NOX4 inhibitors to bridge redox deficits and attenuate early-stage atherogenesis [88].
KLF4 (VSMC Switch)	Master regulator governing the transition of VSMCs from a contractile phenotype to a pro-inflammatory, macrophage-like state.	RNA-based therapeutics (ASOs or siRNAs) designed to reset VSMC plasticity and maintain the structural integrity of the media [91].
Gas6MerTK Axis	Essential molecular eat-me signaling for efferocytosis; its impairment facilitates necrotic core expansion and secondary necrosis.	Utilization of agonists to enhance the clearance of apoptotic foam cells, thereby promoting plaque stabilization and resolving chronic inflammation.
miR-126miR-155	Epigenetic regulators of vascular memory; miR-126 supports repair, while miR-155 promotes aggressive M1 macrophage polarization.	Precision medicine interventions using RNA-based therapies to modulate microRNA expression and suppress the active inflammatory molecular fingerprint [92].
PPAR-ɣ	Modulates the innate immune response by priming monocytes toward pro-resolving M2 macrophage phenotypes.	Therapeutic activation to enhance immune tolerance and promote tissue repair within the aggressive intracranial inflammatory milieu [90].

## Data Availability

No new data were created or analyzed in this study. Data sharing is not applicable.

## Abbreviations

ICAS	Intracranial Atherosclerosis.
ECAS	Extracranial Atherosclerosis.
MCA	Middle Cerebral Artery.
PCA/ACA	Posterior Cerebral Artery/Anterior Cerebral Artery.
HR-VWI	High-Resolution Vessel Wall Imaging.
BAD	Branch Atheromatous Disease.
IPH	Intraplaque Hemorrhage.
KLF2/KLF4	Krüppel-like Factor 2/4.
NO	Nitric Oxide.
eNOS	endothelial Nitric Oxide Synthase.
ROS	Reactive Oxygen Species.
MCP-1	Monocyte Chemoattractant Protein-1.
NF-ɣB	Nuclear Factor kappa-light-chain-enhancer of activated B cells.
DAMPs	Damage-Associated Molecular Patterns.
LDL/oxLDL	Low-density Lipoprotein/Oxidized Low-density Lipoprotein.
RCT	Reverse Cholesterol Transport.
TNF-α/IL-1β/IL-6	Tumor Necrosis Factor-α/Interleukin-1β/Interleukin-6.
MMPs	Matrix Metalloproteinases (specifically MMP-2 and MMP-9).
VSMCs	Vascular Smooth Muscle Cells.
SMA/SMMHC	Smooth muscle α-actin/Myosin Heavy Chain.
BBB	Blood–Brain Barrier.
NVU	Neurovascular Unit.
RNF213	Ring Finger Protein 213.
GWAS	Genome-wide Association Studies.

## References

[1] Xu Y.Y., Li M.L., Gao S., Jin Z.Y., Sun Z.Y., Chen J., Hou B., Zhou H.L., Feng F., Xu W.H. (2017). Etiology of intracranial stenosis in young patients: A high-resolution magnetic resonance imaging study. Ann. Transl. Med..

[2] Gao P., He X., Wang H., Wang T., Wang D., Shi H., Li T., Zhao Z., Cai Y., Wu W. (2025). Stenting Versus Medical Therapy for Symptomatic Intracranial Artery Stenosis: Long-Term Follow-Up of a Randomized Trial. Stroke.

[3] Gong H., Luo J., Xu W., Wang J., Yang R., Yang B., Ma Y., Wang T., Jiao L. (2024). Drug-eluting stents versus bare-metal stents for intracranial atherosclerotic stenosis: A systematic review and meta-analysis. BMJ Open.

[4] Qureshi A.I., Caplan L.R. (2014). Intracranial atherosclerosis. Lancet.

[5] Kamimura T., Okazaki S., Morimoto T., Kobayashi H., Harada K., Tomita T., Higashiyama A., Yoshimoto T., Takahashi J.C., Nakagawara J. (2019). Prevalence of RNF213 p.R4810K Variant in Early-Onset Stroke with Intracranial Arterial Stenosis. Stroke.

[6] Meng F., Xiang H., Huo M., Wu J., Yao W., Wang H. (2026). Research progress in vivo, in vitro, and in silico models of intracranial atherosclerotic stenosis. J. Neurosci. Methods.

[7] Sutton N.R., Malhotra R., St Hilaire C., Aikawa E., Blumenthal R.S., Gackenbach G., Goyal P., Johnson A., Nigwekar S.U., Shanahan C.M. (2023). Molecular Mechanisms of Vascular Health: Insights From Vascular Aging and Calcification. Arterioscler. Thromb. Vasc. Biol..

[8] Sule R.O., Rivera G.D.T., Vaidya T., Gartrell E., Gomes A.V. (2025). Environmental Toxins and Oxidative Stress: The Link to Cardiovascular Diseases. Antioxidants.

[9] Salvagno M., Sterchele E.D., Zaccarelli M., Mrakic-Sposta S., Welsby I.J., Balestra C., Taccone F.S. (2024). Oxidative Stress and Cerebral Vascular Tone: The Role of Reactive Oxygen and Nitrogen Species. Int. J. Mol. Sci..

[10] Yang W.J., Wong K.S., Chen X.Y. (2017). Intracranial Atherosclerosis: From Microscopy to High-Resolution Magnetic Resonance Imaging. J. Stroke.

[11] Chimowitz M.I., Lynn M.J., Derdeyn C.P., Turan N.N., Fiorella D., Lane B.F., Janis L.S., Lutsep H.L., Barnwell S.L., Waters M.F. (2011). Stenting versus Aggressive Medical Therapy for Intracranial Arterial Stenosis. N. Engl. J. Med..

[12] Gao P., Wang T., Wang D., Liebeskind D.S., Shi H., Li T., Zhao Z., Cai Y., Wu W., He W. (2022). Long-Term Effect of Intracranial Stenting vs. Medical Therapy for Symptomatic Intracranial Artery Stenosis: The CASSISS Randomized Clinical Trial. JAMA.

[13] Pu Y., Lan L., Leng X., Wong L.K., Liu L. (2017). Intracranial atherosclerosis: From anatomy to pathophysiology. Int. J. Stroke.

[14] Zhang C., Pu F., Li S., Xie S., Fan Y., Li D. (2013). Geometric classification of the carotid siphon: Association between geometry and stenoses. Surg. Radiol. Anat..

[15] Song J.W., Pavlou A., Xiao J., Kasner S.E., Fan Z., Messé S.R. (2021). Vessel Wall Magnetic Resonance Imaging Biomarkers of Symptomatic Intracranial Atherosclerosis: A Meta-Analysis. Stroke.

[16] Kim B.J., Kim J.S. (2014). Ischemic stroke subtype classification: An asian viewpoint. J. Stroke.

[17] Glagov S., Weisenberg E., Zarins C.K., Stankunavicius R., Kolettis G.J. (1987). Compensatory Enlargement of Human Atherosclerotic Coronary Arteries. N. Engl. J. Med..

[18] Virmani R., Kolodgie F.D., Burke A.P., Farb A., Schwartz S.M. (2000). Lessons from sudden coronary death: A comprehensive morphological classification scheme for atherosclerotic lesions. Arterioscler. Thromb. Vasc. Biol..

[19] Woo N.E., Na H.K., Heo J.H., Nam H.S., Choi J.K., Ahn S.S., Choi H.S., Lee S.K., Lee H.S., Cha J. (2020). Factors for Enhancement of Intracranial Atherosclerosis in High Resolution Vessel Wall MRI in Ischemic Stroke Patients. Front. Neurol..

[20] Wang Y., Zhao X., Liu L., Soo Y.O., Pu Y., Pan Y., Wang Y., Zou X., Leung T.W., Cai Y. (2014). Prevalence and outcomes of symptomatic intracranial large artery stenoses and occlusions in China: The Chinese Intracranial Atherosclerosis (CICAS) Study. Stroke.

[21] Yaghi S., Prabhakaran S., Khatri P., Liebeskind D.S. (2019). Intracranial Atherosclerotic Disease: Mechanisms and Therapeutic Implications. Stroke.

[22] Andelovic K., Winter P., Jakob P.M., Bauer W.R., Herold V., Zernecke A. (2021). Evaluation of Plaque Characteristics and Inflammation Using Magnetic Resonance Imaging. Biomedicines.

[23] Caplan L.R. (1989). Intracranial branch atheromatous disease: A neglected, understudied, and underused concept. Neurology.

[24] Yamamoto Y., Ohara T., Hamanaka M., Hosomi A., Tamura A., Akiguchi I. (2011). Characteristics of intracranial branch atheromatous disease and its association with progressive motor deficits. J. Neurol. Sci..

[25] Holmstedt C.A., Turan T.N., Chimowitz M.I. (2013). Atherosclerotic intracranial arterial stenosis: Risk factors, diagnosis, and treatment. Lancet Neurol..

[26] Wang D., Shang Z.Y., Cui Y., Yang B.Q., Ntaios G., Chen H.S. (2023). Characteristics of Intracranial Plaque in Patients with Non-Cardioembolic Stroke and Intracranial Large Vessel Occlusion. Stroke Vasc. Neurol..

[27] Wang Y., Meng R., Liu G., Cao C., Chen F., Jin K., Ji X., Cao G. (2019). Intracranial atherosclerotic disease. Neurobiol. Dis..

[28] Tekle W.G., Hassan A.E. (2021). Intracranial Atherosclerotic Disease: Current Concepts in Medical and Surgical Management. Neurology.

[29] Deng H., Eichmann A., Schwartz M.A. (2025). Fluid Shear Stress-Regulated Vascular Remodeling: Past, Present, and Future. Arterioscler. Thromb. Vasc. Biol..

[30] Sweet D.R., Padmanabhan R., Liao X., Dashora H.R., Tang X., Nayak L., Jain R., De Val S., Vinayachandran V., Jain M.K. (2023). Krüppel-Like Factors Orchestrate Endothelial Gene Expression Through Redundant and Non-Redundant Enhancer Networks. J. Am. Heart Assoc..

[31] Shinge S.A.U., Zhang D., Din A.U., Yu F., Nie Y. (2022). Emerging Piezo1 signaling in inflammation and atherosclerosis; a potential therapeutic target. Int. J. Biol. Sci..

[32] Shinge S.A.U., Zhang D., Achu Muluh T., Nie Y., Yu F. (2021). Mechanosensitive Piezo1 Channel Evoked-Mechanical Signals in Atherosclerosis. J. Inflamm. Res..

[33] Simo L. (2024). The effects of PM2.5 air pollution on human health: A narrative review with a focus on cerebrovascular diseases. Environ. Dis..

[34] Ritz K., Denswil N.P., Stam O.C.G., van Lieshout J.J., Daemen M.J.A.P. (2014). Cause and Mechanisms of Intracranial Atherosclerosis. Circulation.

[35] Davies P.F. (2009). Hemodynamic shear stress and the endothelium in cardiovascular pathophysiology. Nat. Clin. Pract. Cardiovasc. Med..

[36] Tabas I., Williams K.J., Borén J. (2007). Subendothelial lipoprotein retention as the initiating process in atherosclerosis: Update and therapeutic implications. Circulation.

[37] Kunjathoor V.V., Febbraio M., Podrez E.A., Moore K.J., Andersson L., Koehn S., Rhee J.S., Silverstein R., Hoff H.F., Freeman M.W. (2002). Scavenger receptors class A-I/II and CD36 are the principal receptors responsible for the uptake of modified low density lipoprotein leading to lipid loading in macrophages. J. Biol. Chem..

[38] Yvan-Charvet L., Wang N., Tall A.R. (2010). Role of HDL, ABCA1, and ABCG1 transporters in cholesterol efflux and immune responses. Arterioscler. Thromb. Vasc. Biol..

[39] Libby P., Buring J.E., Badimon L., Hansson G.K., Deanfield J., Bittencourt M.S., Tokgözoğlu L., Lewis E.F. (2019). Atherosclerosis. Nat. Rev. Dis. Primers.

[40] Iliff J.J., Wang M., Liao Y., Plogg B.A., Peng W., Gundersen G.A., Benveniste H., Vates G.E., Deane R., Goldman S.A. (2012). A paravascular pathway facilitates CSF flow through the brain parenchyma and the clearance of interstitial solutes, including amyloid β. Sci. Transl. Med..

[41] Karunakaran D., Geoffrion M., Wei L., Gan W., Richards L., Shangari P., DeKemp E.M., Beanlands R.A., Perisic L., Maegdefessel L. (2016). Targeting macrophage necroptosis for therapeutic and diagnostic interventions in atherosclerosis. Sci. Adv..

[42] Abais J.M., Xia M., Zhang Y., Boini K.M., Li P.L. (2015). Redox Regulation of NLRP3 Inflammasomes: ROS as Trigger or Effector?. Antioxid. Redox Signal..

[43] Latz E., Xiao T.S., Stutz A. (2013). Activation and regulation of the inflammasomes. Nat. Rev. Immunol..

[44] Wei Y., Yang L., Pandeya A., Cui J., Zhang Y., Li Z. (2022). Pyroptosis-Induced Inflammation and Tissue Damage. J. Mol. Biol..

[45] Brosolo G., Da Porto A., Marcante S., Picci A., Capilupi F., Capilupi P., Bulfone L., Vacca A., Bertin N., Vivarelli C. (2023). Lipoprotein(a): Just an Innocent Bystander in Arterial Hypertension?. Int. J. Mol. Sci..

[46] Galis Z.S., Khatri J.J. (2002). Matrix metalloproteinases in vascular remodeling and atherogenesis: The good, the bad, and the ugly. Circ. Res..

[47] Ridker P.M., Everett B.M., Thuren T., MacFadyen J.G., Chang W.H., Ballantyne C., Fonseca F., Nicolau J., Koenig W., Anker S.D. (2017). Antiinflammatory Therapy with Canakinumab for Atherosclerotic Disease. N. Engl. J. Med..

[48] Tardif J.C., Kouz S., Waters D.D., Bertrand O.F., Diaz R., Maggioni A.P., Pinto F.J., Ibrahim R., Gamra H., Kiwan G.S. (2019). Efficacy and Safety of Low-Dose Colchicine after Myocardial Infarction. N. Engl. J. Med..

[49] Hansson G.K., Hermansson A. (2011). The immune system in atherosclerosis. Nat. Immunol..

[50] Ley K. (2020). Role of the adaptive immune system in atherosclerosis. Biochem. Soc. Trans..

[51] Taleb S., Tedgui A., Mallat Z. (2015). IL-17 and Th17 cells in atherosclerosis: Subtle and contextual roles. Arterioscler. Thromb. Vasc. Biol..

[52] Singh B., Li K., Cui K., Peng Q., Cowan D.B., Wang D.Z., Chen K., Chen H. (2022). Defective efferocytosis of vascular cells in heart disease. Front. Cardiovasc. Med..

[53] Kono H., Rock K.L. (2008). How dying cells alert the immune system to danger. Nat. Rev. Immunol..

[54] Zhou R., Yazdi A., Menu P., Tschopp J. (2011). A role for mitochondria in NLRP3 inflammasome activation. Nature.

[55] Mangan M.S.J., Olhava E.J., Roush W.R., Seidel H.M., Glick G.D., Latz E. (2018). Targeting the NLRP3 inflammasome in inflammatory diseases. Nat. Rev. Drug Discov..

[56] Stamatovic S.M., Johnson A.M., Keep R.F., Andjelkovic A.V. (2016). Junctional proteins of the blood-brain barrier: New insights into function and dysfunction. Tissue Barriers.

[57] Iadecola C. (2017). The Neurovascular Unit Coming of Age: A Journey through Neurovascular Coupling in Health and Disease. Neuron.

[58] Sofroniew M.V. (2014). Multiple roles for astrocytes as effectors of cytokines and inflammatory mediators. Neuroscientist.

[59] Bell R.D., Winkler E.A., Sagare A.P., Singh I., LaRue B., Deane R., Zlokovic B.V. (2010). Pericytes control key neurovascular functions and neuronal phenotype in the adult brain and during brain aging. Neuron.

[60] Grigorean V.T., Pantu C., Breazu A., Oprea S., Munteanu O., Radoi M.P., Giuglea C., Marin A. (2026). Mapping the Ischemic Continuum: Dynamic Multi-Omic Biomarker and AI for Personalized Stroke Care. Int. J. Mol. Sci..

[61] Pi H., Wang G., Wang Y., Zhang M., He Q., Zheng X., Yin K., Zhao G., Jiang T. (2024). Immunological perspectives on atherosclerotic plaque formation and progression. Front. Immunol..

[62] Owens G.K., Kumar M.S., Wamhoff B.R. (2004). Molecular regulation of vascular smooth muscle cell differentiation in development and disease. Physiol. Rev..

[63] Grootaert M.O.J., Bennett M.R. (2021). Vascular smooth muscle cells in atherosclerosis: Time for a re-assessment. Cardiovasc. Res..

[64] Deaton R.A., Gan Q., Owens G.K. (2009). Sp1-dependent activation of KLF4 is required for PDGF-BB-induced phenotypic modulation of smooth muscle. Am. J. Physiol. Heart Circ. Physiol..

[65] Hofmann J.J., Iruela-Arispe M.L. (2007). Notch signaling in blood vessels. FEBS Lett..

[66] Shankman L., Gomez D., Cherepanova O., Salmon M., Alencar G.F., Haskins R.M., Swiatlowska P., Newman A.A.C., Greene E.S., Straub A.C. (2015). KLF4-dependent phenotypic modulation of smooth muscle cells has a key role in atherosclerotic plaque pathogenesis. Nat. Med..

[67] Feil S., Fehrenbacher B., Lukowski R., Essmann F., Schulze-Osthoff K., Schaller M., Feil R. (2014). Transdifferentiation of Vascular Smooth Muscle Cells to Macrophage-Like Cells During Atherogenesis. Circ. Res..

[68] Kim J.S., Kim Y.J., Ahn S.H., Kim B.J. (2018). Location of cerebral atherosclerosis: Why is there a difference between East and West?. Int. J. Stroke.

[69] Chen X.Y., Fisher M. (2016). Pathological Characteristics. Front. Neurol. Neurosci..

[70] Su Y., Wang H., Liu H., Tang Y. (2026). Macrophage efferocytosis: Mechanisms and therapeutic opportunities for future cardiovascular diseases. Int. Immunopharmacol..

[71] Mackman N. (2004). Role of tissue factor in hemostasis, thrombosis, and vascular development. Arterioscler. Thromb. Vasc. Biol..

[72] Loscalzo J. (2001). Nitric oxide insufficiency, platelet activation, and arterial thrombosis. Circ. Res..

[73] Ballout A.A., Liebeskind D.S. (2022). Recurrent stroke risk in intracranial atherosclerotic disease. Front. Neurol..

[74] Bang O.Y., Chung J.W., Cha J., Lee M.J., Yeon J.Y., Ki C.S., Jeon P., Kim J.-S., Hong S.C. (2016). A Polymorphism in RNF213 Is a Susceptibility Gene for Intracranial Atherosclerosis. PLoS ONE.

[75] Bagyinszky E., Yang Y., An S.S.A. (2025). Multisystemic Impact of RNF213 Arg4810Lys: A Comprehensive Review of Moyamoya Disease and Associate80d Vasculopathies. Int. J. Mol. Sci..

[76] Tan B.Y.Q., Kok C.H.P., Ng M.B.J., Loong S., Jou E., Yeo L.L.L., Han W., Anderson C.D., Khor C.C., Lai P.S. (2025). Exploring RNF213 in Ischemic Stroke and Moyamoya Disease: From Cellular Models to Clinical Insights. Biomedicines.

[77] Boulanger C.M. (2016). Endothelium. Arterioscler. Thromb. Vasc. Biol..

[78] Mishra A., Malik R., Hachiya T., Jürgenson T., Namba S., Posner D.C., Kamanu F.K., Koido M., Le Grand Q., Shi M. (2022). Stroke genetics informs drug discovery and risk prediction across ancestries. Nature.

[79] Rai H., Parveen F., Kumar S., Kapoor A., Sinha N. (2014). Association of Endothelial Nitric Oxide Synthase Gene Polymorphisms with Coronary Artery Disease: An Updated Meta-Analysis and Systematic Review. PLoS ONE.

[80] Viereck J., Thum T. (2017). Circulating Noncoding RNAs as Biomarkers of Cardiovascular Disease and Injury. Circ. Res..

[81] Damiano G., Rinaldi R., Raucci A., Molinari C., Sforza A., Pirola S., Paneni F., Genovese S., Pompilio G., Vinci M.C. (2024). Epigenetic mechanisms in cardiovascular complications of diabetes: Towards future therapies. Mol. Med..

[82] Jermendy G. (2012). Vascular memory: Can we broaden the concept of the metabolic memory?. Cardiovasc. Diabetol..

[83] Gomyo M., Tsuchiya K., Yokoyama K. (2023). Vessel Wall Imaging of Intracranial Arteries: Fundamentals and Clinical Applications. Magn. Reson. Med. Sci..

[84] Saba L., Cau R., Murgia A., Nicolaides A.N., Wintermark M., Castillo M., Staub D., Kakkos S.K., Yang Q., Paraskevas K.I. (2024). Carotid Plaque-RADS: A Novel Stroke Risk Classification System. JACC Cardiovasc. Imaging.

[85] Van der Toorn J.E., Bos D., Ikram M.K., Verwoert G.C., van der Lugt A., Ikram M.A., Vernooij M.W., Kavousi M. (2022). Carotid Plaque Composition and Prediction of Incident Atherosclerotic Cardiovascular Disease. Circ. Cardiovasc. Imaging.

[86] Liu C., Yang F., Hu Y., Wang L., Li X., Cong H., Zhang J. (2025). The relationships between inflammatory biomarkers, plaque characteristics, and macrophage clusters in coronary plaque: A quantitative assessment of macrophages based on optical coherence tomography. Front. Cardiovasc. Med..

[87] Tanashyan M.M., Raskurazhev A.A., Shabalina A.A., Mazur A.S., Annushkin V.A., Kuznetsova P.I., Illarioshkin S.N., Piradov M.A. (2025). Differential Pattern of Circulating MicroRNA Expression in Patients with Intracranial Atherosclerosis. Biomedicines.

[88] Drummond G.R., Selemidis S., Griendling K.K., Sobey C.G. (2011). Combating oxidative stress in vascular disease: NADPH oxidases as therapeutic targets. Nat. Rev. Drug Discov..

[89] Zheng Q., Zou Y., Teng P., Chen Z., Wu Y., Dai X., Li X., Hu Z., Wu S., Xu Y. (2022). Mechanosensitive Channel PIEZO1 Senses Shear Force to Induce KLF2/4 Expression via CaMKII/MEKK3/ERK5 Axis in Endothelial Cells. Cells.

[90] Bouhlel M.A., Derudas B., Rigamonti E., Dièvart R., Brozek J., Haulon S., Zawadzki C., Jude B., Torpier G., Marx N. (2007). PPARgamma activation primes human monocytes into alternative M2 macrophages with anti-inflammatory properties. Cell Metab..

[91] Kurreck J. (2003). Antisense technologies. Eur. J. Biochem..

[92] Zernecke A., Bidzhekov K., Noels H., Shagdarsuren E., Gan L., Denecke B., Hristov M., Köppel T., Jahantigh M.N., Lutgens E. (2009). Delivery of MicroRNA-126 by Apoptotic Bodies Induces CXCL12-Dependent Vascular Protection. Sci. Signal..

